# Influenza Vaccination Requirements for Health Care Personnel in US Hospitals

**DOI:** 10.1001/jamanetworkopen.2024.16861

**Published:** 2024-06-13

**Authors:** M. Todd Greene, Kathleen A. Linder, Karen E. Fowler, Sanjay Saint

**Affiliations:** 1 Veterans Affairs (VA) Ann Arbor Healthcare System, Ann Arbor, Michigan; 2 Department of Internal Medicine, University of Michigan Medical School, Ann Arbor,; 3 VA/University of Michigan Patient Safety Enhancement Program, Ann Arbor; 4 Infectious Disease Section, VA Ann Arbor Healthcare System, Ann Arbor, Michigan

## Abstract

This survey study assesses changes from 2017 to 2021 in self-reported annual influenza vaccination among workers in nonfederal and Veterans Affairs hospitals.

## Introduction

The Centers for Disease Control and Prevention (CDC) recommends that all US health care personnel (HCP) get vaccinated against influenza annually.^1^ While the CDC and other organizations urge influenza vaccination for all HCP, not all hospitals mandate it. In national surveys of hospitals in 2013 and 2017, influenza vaccination mandates increased from 44% to 69% in nonfederal hospitals, yet remained nearly absent (1% to 4%) in US Department of Veterans Affairs (VA) hospitals.^2^ We sought to ascertain how influenza vaccination requirements for HCP have changed since 2017, especially as mandatory HCP vaccinations were widely discussed during the COVID-19 pandemic.

## Methods

This survey study is part of a multiyear, cross-sectional survey project that asks infection preventionists across the US about health care–associated infection prevention practices.^3^ Using the same national random sample as the 2017 survey wave, beginning in April 2021 we sent a survey (eAppendix in Supplement 1) to 881 nonfederal general medical and surgical hospitals with intensive care units and all 127 VA hospitals. Survey responses were anonymous. Return of the survey served as participant consent. The University of Michigan and VA Ann Arbor Healthcare System Institutional Review Board approved this study.

The survey included the question, Does your hospital mandate health care workers to receive annual influenza vaccination? Declination or opt-out reasons, mask-wearing requirements, and penalties for noncompliance were also assessed. Descriptive statistics were conducted, and aggregate responses from the 2017 and 2021 surveys were compared. χ^2^ tests were used for comparisons. *P* < .05 was considered statistically significant, and hypothesis tests were 2-sided. Analyses were performed in January 2024 using Stata SE 18.0 (StataCorp LLC).

## Results

The overall response rate was 48% (486 respondents from 1008 nonfederal and VA hospitals in the sample). Fifteen respondents (10 from nonfederal, 5 from VA) did not answer the influenza vaccination mandate question and were excluded, leaving 471 respondents (405 from nonfederal, 66 from VA) for the analysis. Questions about influenza vaccination policies for HCP are presented in the Table. Nonfederal hospitals reporting mandatory influenza vaccinations for HCP increased slightly but not significantly,^2^ from 69% (365 of 526) in 2017 to 74% (299 of 405) in 2021 (*P* = .14). In VA hospitals, mandatory influenza vaccinations increased significantly,^2^ from 4% (3 of 73) in 2017 to 96% (63 of 66) in 2021 (*P* < .001).

**Table.** Aspects of Hospital Policies on Annual Influenza Vaccination for Healthcare Personnel, 2021

Survey question	Respondents, No. (%)	
	Nonfederal hospitals	VA hospitals
**All hospitals**		
Does your hospital mandate health care workers to receive annual influenza vaccination?	405	66
Not mandated	12 (3)	1 (2)
Encouraged but not mandated	94 (23)	2 (3)
Mandated with opt-out exemption	299 (74)	63 (96)
Are those who do not receive annual influenza vaccination required to wear a mask when providing patient care during the flu season?	405	66
No	76 (19)	3 (5)
Yes	329 (81)	63 (95)
Are there any penalties for health care workers who are non-compliant with your facility’s policy on influenza vaccination?^a^	364	48
No	140 (38)	33 (69)
Yes	224 (62)	15 (31)
**Only for hospitals with a mandate**		
Which of the following are allowable reasons to opt out or decline vaccination at your hospital (select all that apply)?	299	63
Medical exemption	291 (97)	62 (98)
Religious exemption	244 (82)	60 (95)
No reason required	19 (6)	1 (2)
Other	10 (3)	1 (2)
Are there any penalties for health care workers who are non-compliant with your facility’s policy on influenza vaccination?^b^	260	45
No	67 (26)	30 (67)
Yes	193 (74)	15 (33)

Abbreviation: VA, US Department of Veterans Affairs.

^a^ Fifty-one nonfederal infection preventionists (12%) responded with do not know or did not answer, and 18 VA infection preventionists (29%) responded with do not know.

^b^ Thirty-nine nonfederal infection preventionists (13%) responded with do not know or did not answer, and 18 VA infection preventionists (29%) responded with do not know.

## Discussion

Although mandating influenza vaccination for all HCP remains controversial, this study found a significant increase in VA hospitals requiring vaccinations for HCPs in recent years. Although there was a slight increase in mandates among nonfederal hospitals, mandates in VA hospitals increased substantially and are now nearly universal. This increase follows the implementation of Veterans Health Administration directive 1192.01 in August 2020, which explicitly required VA HCP to either receive influenza vaccination or obtain an exemption annually as a condition of employment.^4^

The findings demonstrate that many hospitals have made institutional commitments to increase vaccination coverage among HCP through mandates. While mandates are a strategy to increase vaccination rates among HCP, they may not be wholly effective in the COVID-19 era. Recent data showed that vaccination rates among HCP, which were increasing before the COVID-19 pandemic (89% in 2017-2018 to 91% in 2019-2020), have steadily decreased during the pandemic (86% in 2020-2021 to 81% in 2022-2023).^5^ Common vaccine hesitancy issues (eg, concerns about safety, mistrust of employers and authorities, and violation of personal autonomy) may have affected this shift.

Study limitations include the moderate response rate, self-reporting bias, and shortcomings of cross-sectional comparisons. Still, the findings demonstrate the value of mandates as a strategy for increasing HCP vaccination rates. Under the principles of nonmaleficence and beneficence, health care institutions and individual HCP have an ethical responsibility to promote universal vaccination of staff^6^ as a means to ensure patient safety.

## Notes

Supplement 1. **eAppendix.** Translating Healthcare-Associated Infection Prevention Research into Practice Survey 2021

Supplement 2.
Data Sharing Statement
