# Supplementary material for: Influenza Vaccination Requirements for Health Care Personnel in US Hospitals
Source: JAMA Netw Open. 2024 Jun 13;7(6):e2416861. doi: 10.1001/jamanetworkopen.2024.16861 (PMC11177160; doi:10.1001/jamanetworkopen.2024.16861)
Supplement: Supplement 2. — Data Sharing Statement [file jamanetwopen-e2416861-s002.pdf]

Data Sharing Statement

Data

Data available: Yes
Data types: Deidentified participant data, Other (please specify)
Additional Information: Survey instrument
How to access data: Data are available upon reasonable request to the corresponding author. These data include anonymous survey responses to questions about infection prevention practices; as such, no patient data were collected.
When available: With publication

Supporting Documents

Document types: None

Additional Information

Who can access the data: Anyone making reasonable request with approved proposed use of data.
Types of analyses: Review and confirmation purposes.
Mechanisms of data availability: After approval of a proposal/request.
